# Melt-Spun Poly(D,L-lactic acid) Monofilaments Containing N,N-Diethyl-3-methylbenzamide as Mosquito Repellent

**DOI:** 10.3390/ma14030638

**Published:** 2021-01-30

**Authors:** Ignatius Ferreira, Harald Brünig, Walter Focke, Regine Boldt, René Androsch, Andreas Leuteritz

**Affiliations:** 1 Leibniz-Institut für Polymerforschung e. V. Dresden (IPF), Hohe Strasse 6, D-01069 Dresden, Germany; ferreira@ipfdd.de (I.F.); bruenig@ipfdd.de (H.B.); boldt@ipfdd.de (R.B.); 2 Institute of Applied Materials and Institute for Sustainable Malaria Control, Department of Chemical Engineering, University of Pretoria, Private Bag X20, Hatfield 0028, South Africa; walter.focke@up.ac.za; 3 Interdisciplinary Center for Transfer-oriented Research in Natural Sciences, Martin Luther University Halle-Wittenberg, D-06099 Halle (Saale), Germany; rene.androsch@iw.uni-halle.de

**Keywords:** poly(lactic acid), DEET, melt spinning, filaments, Malaria, mosquito repellence

## Abstract

Malaria is still a major tropical disease, with Africa particularly burdened. It has been proposed that outdoor protection could aid substantially in reducing the malaria incidence rate in rural African communities. Recently, melt-spun polyolefin fibers containing mosquito repellents have been shown to be promising materials to this end. In this study, the incorporation of N,N‑Diethyl‑3‑methylbenzamide (DEET)—a popular and widely available mosquito repellent—in commercially available, amorphous poly(D,L-lactic acid) (PDLLA) is investigated with the aim of producing biodegradable mosquito-repelling filaments with a reduced environmental impact. It is shown to be possible to produce macroscopically stable PDLLA-DEET compounds containing up to 20 wt.-% DEET that can be melt-spun to produce filaments, albeit at relatively low take-up speeds. A critical DEET content allows for stress-induced crystallization during the spinning of the otherwise amorphous PDLLA, resulting in the formation of α-crystals. Although the mechanical integrity of the filaments is notably impacted by the incorporation of DEET, these filaments show potential as materials that can be used for Malaria vector control.

## 1. Introduction

In 2018, there were an estimated 228 million cases of Malaria worldwide with a co-occurrence of 405,000 deaths of which children under the age of 5 years accounted for more than 65%. Moreover, the region that is the most adversely affected is Africa with a 93% burden of all Malaria cases. Progress has been made from 2010 until 2018 with a decline in the worldwide Malaria incidence rate from 71 to 57 cases per 1000 population at risk. Nevertheless, it is alarming that the incident rate has remained mainly unchanged for the past four years when Africa even saw a consistent increase in Malaria cases from 2014 to 2018 [1].

In a recent study [2], mosquito-repelling socks were developed as a measure of protection for the ankles and feet of people in rural Africa as they are targeted by the malaria vector mosquito, *Anopheles*, in the late afternoon while still active outside [3,4]. The socks were successfully produced from melt-spun fibers that consisted of bicomponent sheath-core structured filaments. N,N‑Diethyl‑3‑methylbenzamide (DEET), the most commonly used topical repellent active [2,5,6,7], was absorbed in poly(ethylene‑co‑vinyl-acetate) (EVA) that served as the core of the filaments while semi-crystalline high density polyethylene (HDPE) served as a release barrier. By incorporating DEET in textiles, the need for frequent reapplication, typical for topical repellents, was eliminated [2,8]. Not only is this more cost effective, but also avoids overuse, reducing the potential skin reactions associated, albeit very rarely, with DEET [8,9].

Although effective, polyolefins are mostly petroleum-based polymers and are nonbiodegradable. The concern is that the urban waste management infrastructure in African countries cannot cope with increased urbanization and that waste management infrastructure in rural areas is largely nonexistent [10]. In Nigeria, a country that is notably affected by Malaria, it is estimated that up to 83% of waste is mismanaged [11]. By considering this, there is a need to develop commercially available alternatives for the core and sheath materials to produce fibers that can provide protection against mosquitos with a reduced environmental impact after use.

The objective of the present work was to explore the possibility of utilizing poly(lactic acid) (PLA) as an alternative as it is a commercially available, biodegradable, compostable, and biocompatible polyester that is obtained from short-term renewable sources [12,13,14,15]. Moreover, the application of PLA ranges from single use packaging to more durable electronic, biomedical, textile, semistructural and automotive applications [16,17,18]. The monomer of PLA has two optically active forms known as L-lactic acid and D-lactic acid that are caused by an asymmetric carbon atom [19]. This renders the possibility of the synthesis of two homopolymers, namely poly(L-lactic acid) (PLLA) and poly(D-lactic acid) (PDLA) as well as D,L-lactic-acid copolymers (PDLLA). Generally, commercially available PLA is often rich in L-isomers containing only a few % D‑isomers as defects [19].

Nonetheless, the D-isomer content is of particular significance as the presence of D-isomers causes a notable deterioration of the crystallization behavior, i.e., a reduction in the crystal melting temperature [19,20], the degree of crystallization and crystal growth rate [21]. Moreover, the higher the D-isomer content, the lower the critical cooling rate to suppress ordering of the melt (30 K·min^−1^ for 0% D-units and 3 K·min^−1^ for 4% D-units) [21]. Ultimately, at 10–12% D-isomer concentrations, or higher, crystallization is completely suppressed in L-rich PDLLA [19,21,22].

In contrast, the incorporation of DEET in PLLA has been shown to increase the rate of crystallization [23,24] and the maximum degree of crystallinity of PLLA [25]. For example, crystallization of pure PLLA could be suppressed at a cooling rate of 5 K·min^−1^, but not in a 50 wt.-% DEET solution [26]. This suggests that a higher critical cooling rate is able to suppress ordering of the melt in the presence of DEET. Moreover, as expected according to the Flory equation describing the equilibrium melting point depression of the polymer [27], a decrease in the crystal melting temperature [25] and crystallization temperature of PLLA have been observed with increasing DEET content.

Besides the depression of the crystallization temperature, or, alternatively in different words, the solid−liquid (S−L) demixing temperature, the amorphous regions of the DEET-PLLA system exhibit liquid−liquid (L−L) demixing and a subambient upper critical solution temperature (UCST). The DEET content of the system investigated in the study of [27] ranged from 70 to 95 wt.‑% and showed a critical temperature of 10 °C occurring at 95 wt.-% DEET. As a result, a DEET-rich PLLA phase and a near pure DEET phase formed [27]. Worth noting, a similar investigation of the L−L demixing behavior has been recently performed on the system PLA/ethyl butylacetylaminopropionate (PLA/IR3535), that is, using a different promising mosquito repellent [28].

To the best of our knowledge, the DEET-PLA phase diagram for polymer-rich compositions is unknown with only a single study performed that investigated samples with 3 and 4.5 wt.-% DEET [23]. Moreover, DEET-containing PLA filaments have not been spun yet by means of melt-spinning. This study aims to address the former as a prerequisite to melt-spinning PLA filaments with a DEET content higher than 4.5 wt.-% for a specific noncrystallizable PDLLA grade, as the crystallization effects lead to nonuniform distribution of DEET due to its enrichment in the amorphous regions and possible L−L demixing. Likewise, the DEET enrichment in amorphous regions caused by crystallization effects could increase the release rate of the DEET.

It was aimed to address three areas of interest in this study, i.e., the solubility of DEET in commercially available noncrystallizable PDLLA, the general melt-spinnability of DEET-PDLLA solutions/compounds, and the characterization of DEET-containing PDLLA monofilaments with varying DEET mass fractions. For characterization, the following methods were considered: thermogravimetric analysis (TGA), differential scanning calorimetry (DSC), X-ray diffraction (XRD), light microscopy, and scanning electron microscopy (SEM).

## 2. Materials and Methods

### 2.1. Materials

A commercially available PLA spinning grade was selected based on the information provided by the manufacturer regarding its crystallizability. To establish the molar mass, size exclusion chromatography (SEC) was employed using chloroform as the mobile phase. The D-unit content was determined by polarimetry where the optical rotation of 1 g dL^−1^ solutions of PLA were measured at a wavelength of 589 nm. The specific optical rotation values for pure PLLA and PDLA were determined as −166° and 166°. The properties of the PLA grade used are given in Table 1. As the D-unit content of the PLA is more than 10%, it can be regarded as amorphous PDLLA.

### 2.2. Preparation of DEET-PDLLA Solutions/Compounds

The as-received PDLLA pellets were dried in a vacuum oven at 40 °C for a minimum of 8 h to remove moisture and were subsequently kept in a desiccator with silica gel. For the determination of the solubility of DEET in the PDLLA, the pellets and DEET were heated to 130 °C in glass vials in a silicon-oil bath on a hot plate to produce samples (≈10 g) with varying DEET content. Three low-DEET-content samples were prepared with 10, 20 and 30 wt.-% DEET as well as four high-DEET-content samples with 70, 80, 90, and 95 wt.-% DEET. For the high-DEET-content samples, a magnetic stirrer at 200 rpm was used as the samples had a low viscosity at 130 °C. It was, however, not possible to make use of the magnetic stirrer in the case of the low-DEET-content samples. These samples were easily pliable at 130 °C and were occasionally compounded manually with a spatula to ensure a homogenous system. Complete dissolution of the pellets in all instances was achieved after 10 to 20 min. Care was a taken to cover the glass vials to minimize the loss of DEET.

The samples were then allowed to cool in the silicon bath to room temperature (21 °C) during which they were briefly removed from the silicon bath at different temperatures to be photographed. The cooling time was determined every 5 K to estimate the cooling rate. In the high-temperature interval (where the cooling rate was the fastest) the cooling rate was approximately 3 K·min^−1^.

For the melt-spinning trials, DEET-PDLLA samples of 20–30 g with DEET contents of 10 and 20 wt.-% were prepared in a similar fashion. Unfortunately, spinning pretrials indicated that spinning the 30 wt.-% samples was not possible. However, the glass vials were exchanged for Petri dishes on a hot plate so that circular discs could be obtained upon cooling. These could then be removed and cut into pellets with pliers for further use. The high surface area of the Petri dishes also allowed for more uniform heating of the samples while the samples were covered with glass discs to minimize the loss of DEET.

### 2.3. Monofilament Spinning

Monofilaments were spun with an in-house designed-and-built melt-spinning device at the Leibniz-Institut für Polymerforschung Dresden e.V. (Dresden, Germany). The device consists of a heated cylinder with a piston with which the volumetric flow rate can be controlled. The piston speed during spinning was kept constant at 10 mm·min^−1^ and by doing so the approximate mass-flow rate was kept constant throughout all spinning trials at roughly 0.8 g min^−1^. For the determination of the spinning range, take-up speeds of up to 1500 m·min^−1^ were investigated. In all instances, the relevant polymer in the cylinder was heated to 190 °C for approximately two minutes before spinning commenced. The holding time was kept to a minimum as release of DEET vapor could be observed at this temperature. All experiments were performed in a room at ambient temperature and no forced convection was used for cooling.

### 2.4. Differential Scanning Calorimetry (DSC)

All DSC trials were performed on a TA Instruments (New Castle, DE, USA) Model Q2000 using nitrogen as purge gas at a flow rate of 50 mL min^−1^. The samples with a mass of about 10 mg, used for the determination of solubility, were transferred to 50-μL aluminum pans and sealed while at 130 °C. The high-DEET-content samples were transferred via a syringe while the low-DEET content samples were transferred via a spatula. The samples were held isothermally at 130 °C for 5 min, before cooling to −20 °C at a rate of 2 K·min^−1^.

For the characterization of the spun monofilaments, samples with a mass of between 1.5 and 3 mg were cut with scissors and sealed in the pans. The samples were held isothermally at −30 °C for 5 min, before they were heated to 230 °C at a rate of 5 K·min^−1^. The results were analyzed with the TA Universal Analysis software. All reported values represent the average of two measurements.

### 2.5. Thermogravimetric Analysis (TGA)

All TGA trials were performed on a TA Instruments (New Castle, DE, USA) Model Q5000. All samples were kept isothermally in 50-μL alumina pans at 22 °C for 10 min, before heating at 10 K·min^−1^ to 500 °C. The heating chamber was purged with nitrogen at a rate of 25 mL min ^−1^. The results were analyzed with the TA Universal Analysis software.

### 2.6. X-ray Diffraction (XRD)

XRD experiments were performed using a Bruker D8 Advance (Billerica, MA, USA) using CuKα radiation (λ = 0.154018 nm). The 2D-diffraction patterns were recorded at room temperature on a 2048 × 2048 pixel Bruker Vantec 500 (Billerica, MA, USA area detector set at a distance of 145.5 mm from the sample. Bundles of filaments were fixed between two clamps. The measurements were performed in transmission mode with a slit size of 0.5 mm perpendicular to the “melt-spin direction”. For phase identification, the 2D patterns were azimuthally integrated to obtain one-dimensional profiles of the scattered intensity as a function of the scattering angle 2Θ = 2.5° to 40°. The XRD analysis was used as a qualitative tool only, i.e., for phase identification and not for quantification of phase fractions or crystal orientations.

### 2.7. Scanning Electron Microscopy

The melt-spun monofilaments were embedded in an epoxy resin and then cut at −120 °C with a Leica UC 7 ultramicrotome (Wetzlar, Germany). The samples were then sputter-coated with 3 nm platinum and imaged with a Zeiss Ultra Plus (Oberkochen, Germany) scanning electron microscope operated at a voltage of 3 kV.

### 2.8. Tensile Tests

The fineness (linear density) of the filaments was determined by weighing bundles of three filaments of 1 m length. The fineness was taken as the average of three of such measurements. Tensile tests were performed at 21 °C on single filaments by using a Zwick Roell Z0,5 Tensile Testing Apparatus (Ulm, Germany). The reported tensile properties represent the average of at least five measurements with a clamp distance of 50 mm and a deformation rate of 200 mm·min^−1^.

## 3. Results

### 3.1. DEET-PLA Phase Behaviour

**Figure 1:**
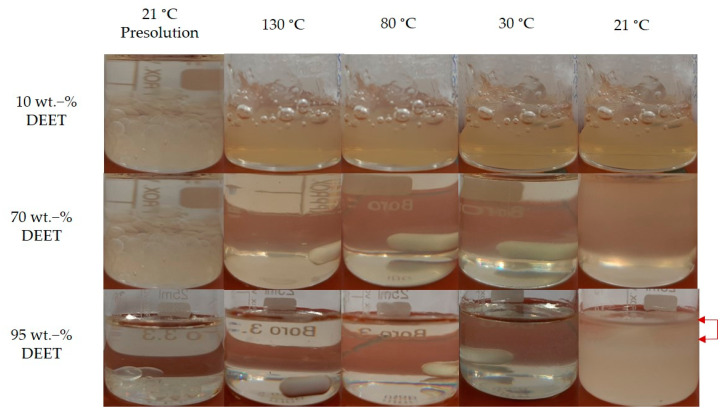
DEET-PDLLA samples with 10, 70, and 95 wt.-% DEET at varying temperatures during cooling.

Figure 1 shows the effect of temperature on the phase behavior of PDLLA-DEET mixtures of varying DEET content. From a macroscopic view, complete dissolution of the PDLLA pellets was observed after about 10 to 20 min at 130 °C (see column 2 in Figure 1). This was the case over the entire investigated DEET-content range. The mixture containing 10 wt.-% DEET remained clear when cooled down even to the lowest temperature considered. This was also true for the 20 and 30 wt.-% DEET samples (not shown here), suggesting the absence of macroscopic phase separation. Contrarily, mixtures containing 70 and 95 wt.-% appeared opaque at 21 °C, indicative of phase separation occurring between 30 and 21 °C [27]. Moreover, bulk phase separation was clearly observed for samples containing 95 and 90 wt.-% (not shown here) at 21 °C, with the formation of a clear, low-viscosity liquid phase (indicated by the red arrows in Figure 1) on top of the viscous phase.

**Figure 2:**
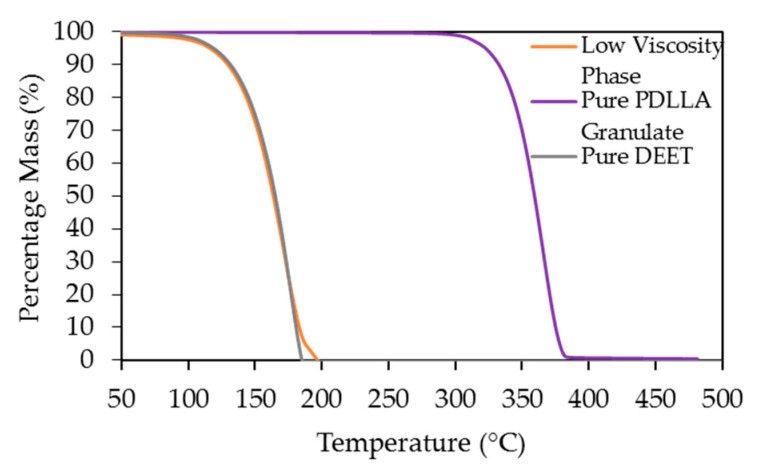
Thermogravimetric analysis (TGA) curves of pure PDLLA, pure DEET and the low viscosity phase separated at 21 °C.

**Figure 3:**
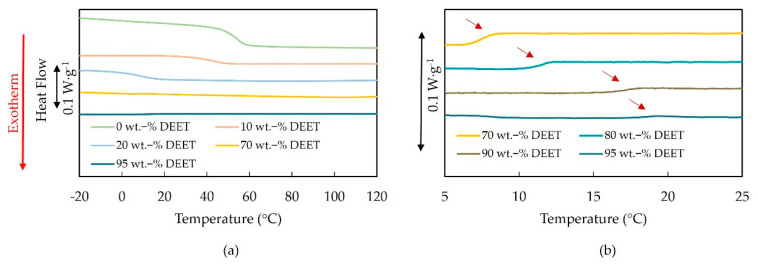
Differential scanning calorimetry (DSC) cooling curves (2 K·min^−1^) of the PDLLA-DEET system at different heat-flow rate scales: (**a**) exhibiting the glass transition of the low-DEET-content samples; (**b**) exhibiting the demixing peaks of high-DEET samples.

The composition of the low-viscosity liquid phase (separated at 21 °C), was determined by TGA analysis. This is shown in Figure 2 where pure DEET is also included as a reference. It was clear that the low-viscosity phase at this temperature approached pure DEET in nature and this was in good agreement with previous findings [27,29]. However, these results do not indicate whether the phase separation at these high DEET contents was due to crystallization (S−L demixing) followed by L−L demixing or only due to L−L demixing. To shed light on this matter, the DSC cooling curves for the solutions and PDLLA without DEET (cooling rate 2 K·min^−1^) are shown in Figure 3a.

No exothermic crystallization event could be observed for any sample within the investigated temperature range of 130 °C to −20 °C, with particular reference to the range between 30 °C and 21 °C. This was significant since it confirmed that the higher opacity of the samples with 70–90 wt.-% DEET at 21 °C was not a result of S−L demixing, but only L−L demixing. Moreover, it showed that if crystallization is theoretically possible, then it was completely suppressed at a cooling rate of 2 K min^‑1^, regardless of the DEET content. This was a result of the high D-unit content of 11.3% of the present PDLLA as the crystallization of PDLLA with a 4% D-unit content was only suppressed at 3 K·min^−1^. In theory, even a higher cooling rate would be required to suppress crystallization of PDLLA in the presence of DEET [24].

L−L demixing was further evidenced by the detection of small heats of dissolution for these solutions, shown in Figure 3b where the same cooling curves as in Figure 3a are shown, but with magnified scale. The lower the DEET content, the lower the demixing temperature—in line with previous investigations [27,29]. The demixing temperatures for the 90 wt.-% (18 °C) and 95 wt.-% (19 °C) DEET solutions, were in the near vicinity of 21 °C where bulk phase separation had been observed. However, the demixing temperature of the 70 wt.-% solution (6 °C) was far lower than the 21 °C at which the opacity had already changed. A possible explanation is the difference in cooling rates between the two methods. The cooling rate for the DSC measurement was constant at 2 K min^‑1^ for the entire range, in contrast to the cooling rate for the solutions in Figure 2 that decreased with decreasing temperature. In the latter case, the average cooling rate between 30 and 21 °C of the solutions could be approximated as 0.3 K·min^−1^.

Moreover, glass transitions were only observable for the PDLLA without DEET (onset 58 °C) and the solutions with 10 wt.-% (onset 33 °C), 20 wt.-% (onset 16 °C) and 30 wt.-% DEET (onset −9 °C). The only slight broadening of the glass transition temperature (Tg) with DEET, together with the continuous decrease of the Tg with increasing DEET confirmed that DEET and PLA are miscible at 10, 20 and 30 wt.-% [23,30,31]. Nevertheless, some microscopic heterogeneities may exist.

**Figure 4:**
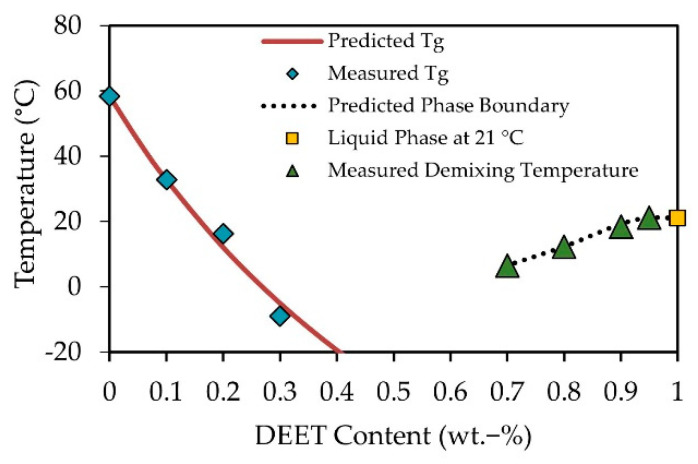
Phase diagram of the polymer/solvent system PDLLA/DEET containing information about glass transition temperatures in low-DEET-content samples (blue diamond symbols) and temperatures of demixing of high-DEET-content samples (green triangle symbols).

In Figure 4, the Tgs (blue diamond symbols) and demixing temperatures (green triangle symbols) are shown as well as the composition of the low viscosity phase at 21 °C (yellow square symbol). The predicted theoretical Tgs, by fitting the Gordon−Taylor Equation, are given by the solid red line.

The phase diagram is in good agreement with the phase diagram proposed in reference [27]. The amorphous PLA exhibited a UCST at approximately 21 °C and 95 wt.-% DEET with the formation of a DEET-rich PDLLA phase and near pure DEET phase. It should, however, be noted that the critical temperature was notably higher than the reported 10 °C. Nonetheless, of significance for the purposes of melt spinning DEET-containing PDLLA filaments, it has been shown that a macroscopically homogenous PDLLA-DEET phase exists at DEET contents of up to at least 20 wt.-% where the DEET has a significant impact on the glass transition temperature.

### 3.2. Spinnability of PDLLA Monofilaments with Varying DEET Content

**Figure 5:**
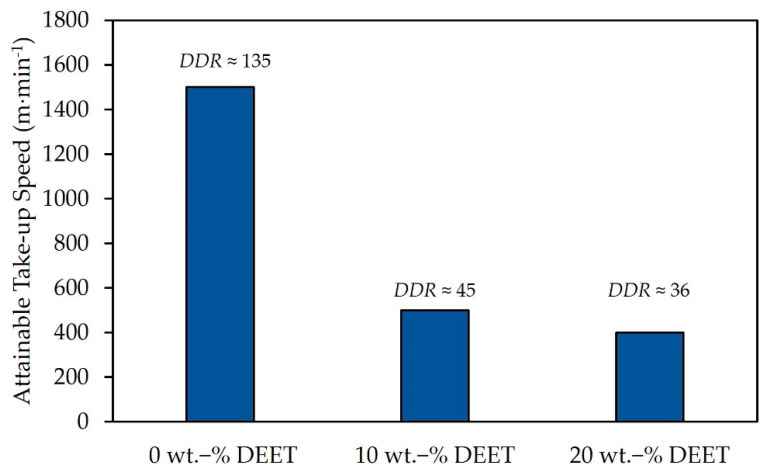
Attainable take-up speeds for melt-spun DEET-containing PDLLA monofilaments as a function of DEET content.

In Figure 5, the attainable take-up speeds of the PDLLA monofilaments with varying DEET content are shown together with the approximated associated draw-down ratio (*DDR*). Note that the maximum take-up speed investigated did not exceed 1500 m·min^−1^.

The pure PDLLA monofilaments could be spun at the maximum take-up speed investigated, i.e., 1500 m·min^−1^. Conversely, the addition of DEET resulted in a notable decrease of the attainable take-up speed to 500 m·min^−1^ and 400 m·min^−1^ for 10 wt.-% and 20 wt.-% DEET, respectively. This shows that DEET-containing PDLLA monofilaments can indeed be produced, although only at relatively low take-up speeds compared to the general standard of at least 1000 m·min^−1^ [32]. This is likely due to the marked decrease in viscosity due to DEET addition. For comparison with samples containing 20 wt.-% PDLLA, monofilaments were spun at 500 m·min^−1^ in all other instances for further analyses.

### 3.3. Semicrystalline Morphology

**Figure 6:**
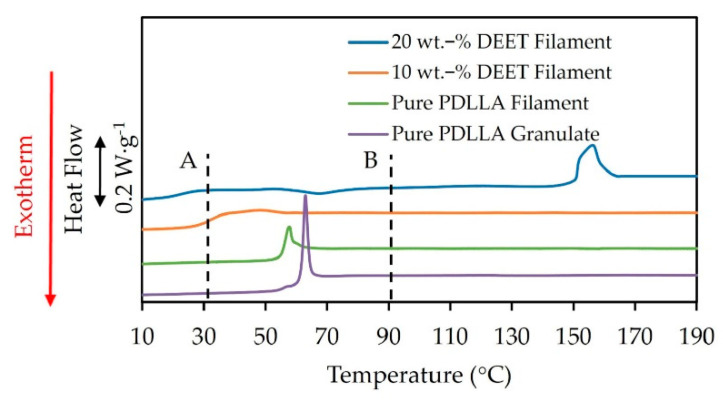
DSC heating scans, recorded at a heating rate of 5 K·min^−1^, of PDLLA monofilaments containing different amounts of DEET.

DSC heating scans, recorded at a rate of 5 K·min^−1^, of PDLLA monofilaments with varying DEET content are shown in Figure 6. As reference, the heating scan of as-received PDLLA granulate without DEET is also included. Note that the slopes were adjusted manually to fit the general trend of increasing heat capacity (*Cp*) with increasing temperature.

The PDLLA granulate and the PDLLA monofilaments without DEET exhibited only a single glass transition temperature at 55 °C that was superimposed by an endothermic enthalpy-recovery peak, which was expectedly greater for the PDLLA granulate [25,33]. The absence of cold crystallization and melting upon heating implies that neither crystals nor nuclei formed during the spinning process. By considering the high cooling rates that are typical of the melt-spinning process [34], it is in line with the findings in Section 3.1 where a cooling rate as low as 2 K·min^−1^ had already completely suppressed crystallization. However, the melt-spinning process is typically characterized by high elongational strain rates [35] that have been shown to notably increase the degree of crystallization by stress-induced crystallization [36]. Nonetheless, it seems that the high D-unit content prevented not only thermally induced crystallization but also stress-induced crystallization in the pure PDLLA filaments and when DEET was incorporated at a concentration of 10 wt.-%.

In contrast, at 20 wt.-% DEET, an exothermic cold-crystallization peak at 68 °C and endothermic melting peak at 157 °C, were observed. The heat of melting exceeded the heat of cold crystallization as shown in Table 2, indicating that minor crystallization took place during the spinning process regardless of the high cooling rate. Upon heating, additional nuclei continued to grow during the cold-crystallization event. Note that no crystallization was observed for the 20 wt.-% DEET solution cooled at 2 K·min^−1^ in Section 3.1. This implies that the high elongational strain rate during the spinning process plays a determinant role in the crystallization process of these filaments. A possible explanation is that the DEET allows for a greater mobility of PDLLA chain segments, thus allowing their transition to an ordered state more easily when drawn during the melt-spinning process. This is in agreement with the study of [25] that reported that no crystallization had occurred during electrospinning of pure PLLA, but the incorporation of DEET markedly increased crystallization.

**Figure 7:**
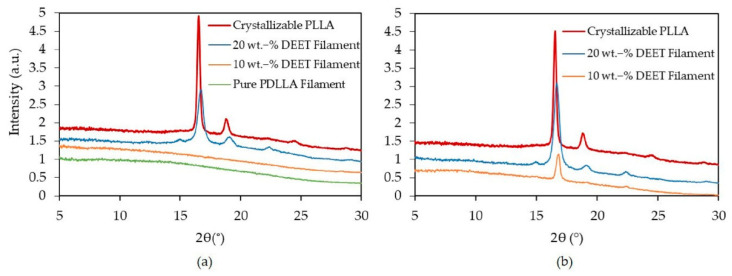
XRD-scans of PDLLA monofilaments with varying DEET content (**a**) after spinning and (**b**) after additional subsequent annealing at 80 °C for 1 h.

XRD measurements, shown in Figure 7, were performed to confirm the presence of crystals and to establish the crystal form of the monofilaments with 20 wt.-% DEET. To replicate the effect of cold crystallization during heating, the filaments were annealed in an oven for 1 h at 80 °C. The XRD scans of the filaments after spinning (Position A in Figure 6) are shown in Figure 7a whereas the scans of the annealed filaments are shown in Figure 7b (Position B in Figure 6). Crystallizable PLLA annealed at 80 °C is also included as reference in both instances.

Crystallizable PLLA forms α’-crystals at temperatures below 120 °C. This morphology is a less stable crystal structure with a looser chain packing compared to the stable α-form that forms at temperatures exceeding 100 °C [19,37,38,39,40,41]. The presence of α’-crystals was confirmed by the intense peaks at 16.6° 2θ (planes (110)/(200)) and 19.1° (planes(203)/(113)) as well as the absence of the (211) peak at 22.4° [42,43].

From Figure 7a, a halo is visible for the PDLLA monofilaments containing 0 and 10 wt.-% DEET. This confirms that these filaments did at most undergo miniscule crystallization during the spinning process, being in line with the DSC results. Conversely, the PDLLA monofilaments with 20 wt.‑% DEET showed intense peaks at 16.8° (planes (110)/(200)) and 19.1° (planes (203)/(113)), i.e., a general shift to a higher 2θ compared to the reference. This, and a peak at 22.4° (plane (211)) confirmed the presence of the α-crystal form [42,43].

In Figure 7b, the annealed PDLLA monofilaments containing 20 wt.-% DEET exhibited an intensification of the existing peaks. This implies that the cold-crystallization event observed during heating leads to the continued formation and growth of α-crystals. Significantly, the emergence of the (110)/(200) peak at 16.8° 2θ in the case of 10 wt.-% DEET filaments was also observed when annealed. Since no cold crystallization was observed for the same filaments during the DSC heating scan, it can be concluded that the DSC heating rate had also played a determinant role.

By establishing the nature of the crystals that formed in the 20 wt-% DEET, it was possible to estimate the degree of crystallinity at points A and B in Figure 6. The bulk heat of melting (∆H°m_α) of α-crystals is 135 J g^−1^ and 91 J g^−1^ at 157 °C and 68 °C respectively, as estimated from [43]. This resulted in crystallinities at points A and B (in Figure 6) of 6 and 8%, respectively, for the filaments with 20 wt.-% DEET.

In summary, for the given DEET-PDLLA compounds containing 10 and 20 wt.-% DEET, crystallization was not observed when cooling at 2 K·min^−1^. However, if the 20 wt.-% solution was spun, α-crystals formed. This occurred regardless of the high cooling rate during the melt-spinning process and suggested that the high elongational strain rate of the process was a decisive factor, promoting stress-induced crystallization. Nevertheless, this was not observed for lower DEET contents, implying that a critical DEET content allows for increased segmental mobility for the chains to assemble in an ordered state during the spinning process.

### 3.4. DEET Content and Distribution

**Figure 8:**
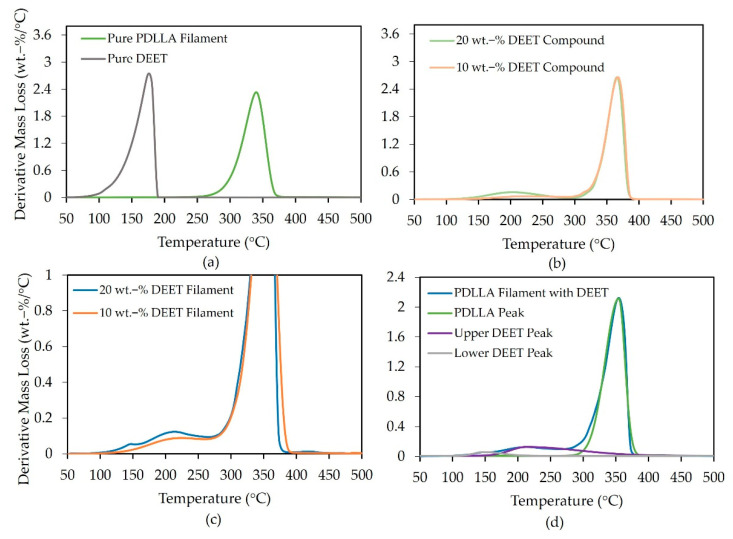
TGA derivative-mass-loss curves of DEET and PDLLA monofilaments (**a**) without DEET, (**b**) PDLLA-DEET compounds with varying DEET content, (**c**) PDLLA monofilaments with varying DEET content and (**d**) a representative deconvolution curve of 20 wt.-% DEET monofilaments.

Derivative TGA-mass-loss curves of DEET and the PDLLA filaments are given in Figure 8a–d. From Figure 8a, where no DEET was present in the filaments, it was observed that the PDLLA filaments have a thermal degradation peak at 344 °C, with completion near 380 °C. The onset of mass loss for pure DEET was at around 95 °C with a peak at 176 °C and completion by 190 °C.

PDLLA granulate-DEET compounds with varying fractions of DEET in Figure 8b, showed two distinct mass-loss steps. The higher mass-loss event corresponded to the thermal degradation of the PDLLA. The onset of the lower mass-loss event was at 145 °C with a peak at 215 °C. Although, at a significantly higher temperature, this event scaled with the DEET content. This could be beneficial for the prospect of using the filaments for the controlled release of DEET as it suggests delayed evaporation of the DEET when dissolved in the PDLLA.

Spun PDLLA-DEET compounds with varying fractions of DEET in Figure 8c exhibited the same two mass-loss events. Notably, in the case of 20 wt.-% DEET filaments, a third peak at 145 °C was observed that corresponded to the DEET-mass-loss peak that was reported in [25]. It appears that the lower-temperature peak was not directly dependent on the mass fraction of DEET, but rather on the presence of a crystalline phase as this was the only case where a crystalline phase was detected in the PDLLA filaments. This phenomenon is, however, not yet fully understood.

A representative curve of the deconvoluted peaks to estimate the amount of DEET in the filaments is shown in Figure 8d, and estimated DEET mass fractions are given in Table 3.

The experimentally determined DEET mass fractions scale with the intended theoretical mass fractions. The amount of DEET lost during the spinning process, however, cannot be accurately estimated from the current data. Nevertheless, it has been proven that DEET-containing filaments can be melt-spun while retaining most of the initial DEET during the spinning process.

### 3.5. Filament Morphology and Size

**Figure 9:**
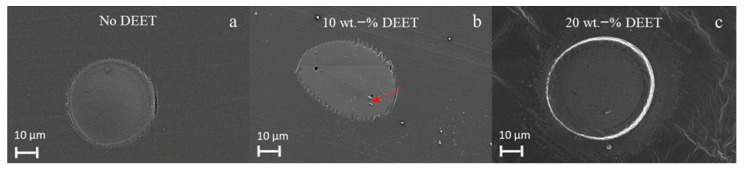
SEM images of PDLLA monofilaments with no DEET (**a**), 10 wt.-% DEET (**b**) and 20 wt.-% DEET (**c**).

In Figure 9, SEM images of the PDLLA filaments with varying DEET content are shown. Circular filaments within the micrometer-range were observed with an outer boundary ring that was due to the preparation method and should not be confused with a sheath boundary. In some instances, where DEET was incorporated, globular structures (indicated by the red arrow) were also observed. This is possibly due to the evaporation of the DEET during the spinning process at 190 °C as the TGA results indicated that the onset DEET mass loss in PDLLA had occurred already at a temperature as low as 145 °C.

The average filament diameter (*Ø*), determined by optical microscopy, and the linear density (*F*) of the filaments are given in Table 4. There was a definite increase in the filament diameter with increasing DEET content that could be attributed to the greater free volume. It should be kept in mind, though, that the PDLLA filaments with 20 wt.-% DEET had also been spun at a slightly lower take-up speed. Furthermore, the incorporation of DEET led to a significant increase in the linear density of the filaments. This marked higher linear density of the PDLLA filaments with 20 wt.-% DEET may also be due to the existence of the crystalline phase.

### 3.6. Filament Mechanical Integrity

**Figure 10:**
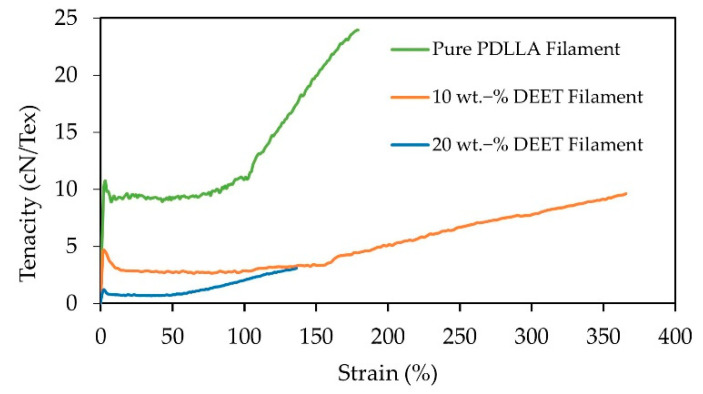
Representative stress−strain curves of PDLLA filaments with varying DEET content.

Representative stress−strain curves of the PDLLA filaments with varying DEET content are presented in Figure 10. In all cases, a clear yield point was observed that was followed by notable plastic deformation and strain hardening before failure. The prominent plastic deformation region was expected, as the filaments were spun at a relatively low take-up speed of 400 m·min^−1^ (20 wt.‑% DEET) or 500 m·min^−1^. A lengthening of the plastic deformation region, as well as an increase in the elongation at break, was observed for the PDLLA filaments with 10 wt.-% compared to the DEET-free PDLLA filaments. This suggested the DEET enabled a higher degree of chain orientation during the mechanical drawing, attributed to more freedom for the molecular chains to rearrange. However, this was not true for the PDLLA filaments with 20 wt.-% DEET. Substantially, this was also the case when there was crystallization in the presence of DEET. Therefore, there are two opposing factors, i.e., an increase in ductility due to the increased chain mobility in the amorphous phase vs. the rigidity of the crystalline phase. From Table 5, it can be seen that the addition of DEET led to a significant decrease in the yield strength, which was in line with the increased mobility of the PLA chains, as well as a reduction of the ultimate strength and the elastic modulus. Considering the significant impact that the incorporation of DEET has on the mechanical integrity of the filaments, it is proposed to introduce a sheath polymer that could potentially allow higher attainable take-up speeds and simultaneously act as a tailorable release barrier to the DEET. Moreover, the postdrawing of the filaments could also aid in improving the knittability of these filaments.

## 4. Conclusions

DEET can be incorporated into commercially available noncrystallizable PDLLA to produce compounds containing 10 wt.-% and 20 wt.-% DEET that are macroscopically stable. It has been shown that it is possible to produce DEET-containing PDLLA monofilaments in the micrometer-range from these compounds by melt spinning. The DEET content, however, greatly limits the attainable take-up speed. Moreover, DEET plays a critical role in enabling stress-induced crystallization of α-crystals during the spinning process that is amorphous under the same conditions without the presence of DEET. The degree of crystallinity of 6% is albeit relatively low in the case of 20 wt ‑% DEET monofilaments. These results show promise that it is possible to produce biodegradable mosquito-repelling filaments from a commercially available PDLLA grade that can serve as an environmentally alternative to existing petroleum-based filaments, however their effectiveness at the current DEET loading still needs to be determined. Moreover, it is proposed to introduce a sheath polymer to improve the mechanical integrity of the filaments that can potentially simultaneously aid in tailoring the release rate of the DEET.

## Figures and Tables

**Table 1:** Molar percentage of D-units (*X_D_*), mass-average molar mass (*M_w_*), number-average molar mass (*M_n_*), and polydispersity (*D*) of PLA used in the present work.

*X_D_* (%)	*M_w_* (kDa)	*M_n_* (kDa)	*D* (-)
11.3	147.0	46.8	3.15

**Table 2:** Summary of thermal events for pure PDLLA granulate and PDLLA monofilaments with varying DEET content.

	Pure PDLLA Granulate	Pure Spun PDLLA	Spun PDLLA with 10 wt.-% DEET	Spun PDLLA with 20 wt.-% DEET
Tg onset (°C)	53.6 ± 0.6	55.1 ± 3.9	27.4 ± 1.6	18.5 ± 0.1
CC peak (°C)	-	-	-	67.5 ± 0.1
Melting peak (°C)	-	-	-	156.4 ± 0.5
ΔH_m_ (J/g)	-	-	-	11.4 ± 0.1
ΔH_cc_ (J/g)	-	-	-	2.3 ± 0.4

**Table 3:** Estimated DEET content of PDLLA monofilaments with varying DEET content.

Description	PDLLA with 10 wt.-% DEET	PDLLA with 20 wt.-% DEET
Experimental DEET content	12.8	17.8
Theoretical DEET content	10.0	20.0

**Table 4:** Dimensions of the PDLLA monofilaments with varying DEET mass fractions.

Property	PDLLA with 0 wt.‑% DEET	PDLLA with 10 wt.‑% DEET	PDLLA with 20 wt.‑% DEET
*Ø* (μm)	30.7 ±0.8	38.9 ± 2.8	49.0 ± 7.5
F (dTex)	6.3	12.7	26.3

**Table 5:** Tensile properties PDLLA filaments with varying DEET content.

Tensile Property	PDLLA with 0 wt.‑% DEET	PDLLA with 10 wt.‑% DEET	PDLLA with 20 wt.‑% DEET
Elastic modulus (cN/Tex)	628.5 +/− 53.2	368.8 +/− 9.1	129.8 +/− 29.2
Ultimate strength (cN/Tex)	24.7 +/− 0.6	9.3 +/− 0.3	3.6 +/− 0.7
Elongation at break (%)	181.6 +/− 8.9	376.0 +/− 13.2	148.1 +/− 20.1

## Data Availability

The data presented in this study are available on request from the corresponding author.
